# Consensus protocols for the diagnosis and management of the hereditary autoinflammatory syndromes CAPS, TRAPS and MKD/HIDS: a German PRO-KIND initiative

**DOI:** 10.1186/s12969-020-0409-3

**Published:** 2020-02-17

**Authors:** Sandra Hansmann, Elke Lainka, Gerd Horneff, Dirk Holzinger, Nikolaus Rieber, Annette F. Jansson, Angela Rösen-Wolff, Gabi Erbis, Martina Prelog, Juergen Brunner, Susanne M. Benseler, Jasmin B. Kuemmerle-Deschner

**Affiliations:** 1 Department of Pediatric Rheumatology, autoinflammation reference centre Tuebingen (arcT), University Children’s Hospital Tuebingen, Tuebingen, Germany; 2 Department of Pediatric Rheumatology, University Children’s Hospital Essen, Essen, Germany; 3 Department of Pediatrics, Asklepios Clinic Sankt Augustin GmbH, Sankt Augustin, Germany; 4 Department of Pediatric Hematology-Oncology, University of Duisburg-Essen, Essen, Germany; 5 Department of Pediatrics, Kinderklinik Muenchen Schwabing, Klinikum Schwabing, StKM GmbH und Klinikum rechts der Isar, Technical University of Munich, Munich, Germany; 6 Department of Pediatrics I, University Children’s Hospital Tuebingen, Tuebingen, Germany; 7 Division of Pediatric Rheumatology and Immunology, Dr. von Hauner Children’s Hospital, University Hospital Munich, Munich, Germany; 8 Department of Pediatrics, University Hospital Carl Gustav Carus, TU Dresden, Dresden, Germany; 9 Department of Pediatrics, Pediatric Rheumatology and Special Immunology, University Hospital Wuerzburg, Wuerzburg, Germany; 10 Department of Pediatrics, Medical University Innsbruck, Innsbruck, Austria; 11 Rheumatology, Department of Pediatrics, Alberta Children’s Hospital, Alberta Children’s Hospital Research Institute, Cumming School of Medicine, University of Calgary, Calgary, Canada

**Keywords:** Autoinflammatory diseases, Treat-to-target, Consensus treatment plans, Management, Comparative effectiveness

## Abstract

**Background:**

Rare autoinflammatory diseases (AIDs) including Cryopyrin-Associated Periodic Syndrome (CAPS), Tumor Necrosis Receptor-Associated Periodic Syndrome (TRAPS) and Mevalonate Kinase Deficiency Syndrome (MKD)/ Hyper-IgD Syndrome (HIDS) are genetically defined and characterized by recurrent fever episodes and inflammatory organ manifestations. Early diagnosis and early start of effective therapies control the inflammation and prevent organ damage. The PRO-KIND initiative of the German Society of Pediatric Rheumatology (GKJR) aims to harmonize the diagnosis and management of children with rheumatic diseases nationally. The task of the PRO-KIND CAPS/TRAPS/MKD/HIDS working group was to develop evidence-based, consensus diagnosis and management protocols including the first AID treat-to-target strategies.

**Methods:**

The national CAPS/TRAPS/MKD/HIDS expert working group was established, defined its aims and conducted a comprehensive literature review synthesising the recent (2013 to 2018) published evidence including all available recommendations for diagnosis and management. General and disease-specific statements were anchored in the 2015 SHARE recommendations. An iterative expert review process discussed, adapted and refined these statements. Ultimately the GKJR membership vetted the proposed consensus statements, agreement of 80% was mandatory for inclusion. The approved statements were integrated into three disease specific consensus treatment plans (CTPs). These were developed to enable the implementation of evidence-based, standardized care into clinical practice.

**Results:**

The CAPS/TRAPS/MKD/HIDS expert working group of 12 German and Austrian paediatric rheumatologists completed the evidence synthesis and modified a total of 38 statements based on the SHARE recommendation framework. In iterative reviews 36 reached the mandatory agreement threshold of 80% in the final GKJR member survey. These included 9 overarching principles and 27 disease-specific statements (7 for CAPS, 11 TRAPS, 9 MKD/HIDS). A diagnostic algorithm was established based on the synthesized evidence. Statements were integrated into diagnosis- and disease activity specific treat-to-target CTPs for CAPS, TRAPS and MKD/HIDS.

**Conclusions:**

The PRO-KIND CAPS/TRAPS/MKD/HIDS working group established the first evidence-based, actionable treat-to-target consensus treatment plans for three rare hereditary autoinflammatory diseases. These provide a path to a rapid evaluation, effective control of disease activity and tailored adjustment of therapies. Their implementation will decrease variation in care and optimize health outcomes for children with AID.

## Background

Autoinflammatory diseases (AIDs) comprise a heterogeneous group of genetically and/or clinically defined conditions characterized by unprovoked systemic inflammation causing fever episodes and inflammatory organ manifestations [1, 2]. Mutations in inflammatory genes result in constitutionally raised levels of secreted pro-inflammatory cytokines such as Interleukin-1 (IL-1) [3]. AIDs carry a significant mortality and morbidity including failure to thrive, hearing loss and renal failure. While Familiar Mediterranean Fever (FMF) can be found with a high prevalence of 1/1000 in several Mediterranean populations [4], other AIDs including Cryopyrin-Associated Periodic Syndrome (CAPS) [5, 6], TNF Receptor-Associated Periodic Syndrome (TRAPS) [7] and Mevalonate Kinase Deficiency Syndrome (MKD)/ Hyper-IgD Syndrome (HIDS) [8] are rare and have an incidence of less than 1/1.000.000 [9].

Diagnosing a rare AID mandates the recognition of characteristic clinical findings. Diagnostic criteria are currently only available for CAPS [10]; classification criteria have recently been validated for FMF, TRAPS, MKD, CAPS and PFAPA addressing genetic and clinical criteria [11, 12]. A molecular genetic confirmation of a rare AID is desirable, yet not always possible. Increased awareness of clinicians and access to genetic testing has significantly reduced the delay to diagnosis in many countries [4]. However, the variability of disease activity has not been addressed in diagnostic or classification criteria. Atypical clinical phenotypes and late-onset subtypes associated with somatic mutations can be seen [13].

The optimal day-to-day management of rare AIDs remains challenging. Traditional immunosuppressive medications such as prednisone often have limited effect and may even shorten intervals between flares [14]. Targeted inhibition of specific pro-inflammatory cytokines is commonly required. Early effective treatment of the underlying systemic inflammatory process prevents fever episodes and organ damage [15, 16]. Significant variations in care impact on patient outcomes [17]. Access to effective medications varies between countries; coverage of the frequently prohibitive drug costs remains a major barrier [18].

In 2015, the Single Hub and Access point for pediatric Rheumatology in Europe (SHARE) initiative set out to improve the management of patients with rheumatic diseases including AIDs [19]. SHARE investigators synthesized the published evidence and developed management recommendations. Important guiding principles were established; optimal treatment selection and monitoring concepts were defined. However, the effective implementation of evidence-based treatment recommendations into clinical practice mandates development of consensus protocols. Entry criteria for distinct levels of disease activity, risk factors for adverse outcomes and sensible timelines for evaluating outcomes have to be defined.

The PRO-KIND (PROjekte zur Klassifikation, Überwachung und Therapie in der KINDerrheumatologie, Projects for the classification, monitoring and therapy in pediatric rheumatology) initiative is a sub-committee of the German Society for Pediatric Rheumatology (GKJR). The national PRO-KIND initiative addresses the urgent need to improve quality of care and outcome of children and adolescents living with rheumatic conditions by standardization and effective implementation of evidence-based therapies and thereby decreasing variation in care [20–22].

The aims of the PRO-KIND initiative for CAPS/TRAPS/MKD/HIDS were 1) to establish consensus-based statements for the diagnosis and management and 2) to establish national treat-to-target consensus treatment plans (CTPs) for CAPS, TRAPS and MKD/HIDS suitable for implementation. A separate working group for FMF was established [23].

## Methods

### PRO-KIND initiative and CAPS/TRAPS/MKD/HIDS expert working group

**Fig. 1:**
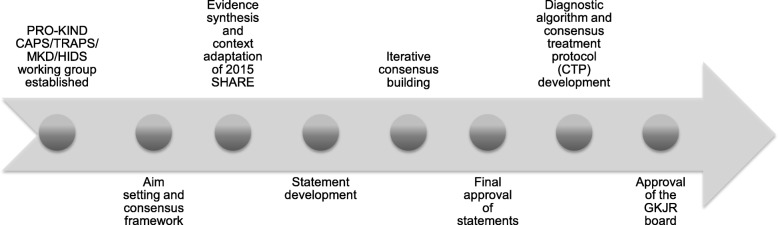
Projects for the classification, monitoring and therapy in pediatric rheumatology (PRO-KIND) CAPS/TRAPS /HIDS/MKD working group: Evidence-based statement and consensus treatment plan (CTP) development. The PRO-KIND CAPS/TRAPS /HIDS/MKD working group of the German Society of Pediatric Rheumatology (GKJR) started in October 2015. Evidence-based statements were crafted anchored in the 2015 SHARE recommendations and vetted in iteratively online surveys and telephone conferences. Statements and consensus treatment plans for CAPS, TRAPS and HIDS/MKD were established and vetted by the GKJR membership

The PRO-KIND initiative started in January 2015 aiming to standardize the diagnosis and management of children with rheumatic diseases nationally. All members of the GKJR were invited to participate in the process. A priori, a unified formal PRO-KIND framework and multi-step process for the development of consensus-based protocols was established. The PRO-KIND working group for the rare hereditary autoinflammatory syndromes CAPS, TRAPS and MKD/HIDS was established in February 2015. The speaker (JKD) and the coordinator (EL) were selected. The approach of the PRO-KIND CAPS/TRAPS/MKD/HIDS working group included 1) evidence synthesis including published recommendations and context adaptation, 2) statement development 3) iterative consensus building and final approval of statements and 4) diagnostic algorithm and treat-to-target CTP development (see Fig. 1).

### Evidence synthesis, context adaptation and statement development

Members of the PRO-KIND working group have previously led (JKD) or participated (SMB) in the Europe-wide SHARE initiative and developed evidence-based recommendations for the diagnosis and management of autoinflammatory diseases [19]. These had synthesized the published evidence until June 2013. Therefore, the PRO-KIND working group anchored the PRO-KIND statements in the SHARE recommendations and reviewed the relevant evidence of studies published July 2013 until January 2018. The SHARE recommendations were modified based on new evidence and cultural context and adjusted, where appropriate. Evidence levels and strength of recommendations were adapted accordingly. The Oxford Centre for Evidence-based Medicine levels of evidence and grades of recommendation were utilized to support the evidence for each statement [24]. Modified recommendations were drafted and iteratively discussed, adjusted and vetted in regular telephone conferences. All members of the PRO-KIND working group were asked to participate in seven teleconferences/face-to-face meetings and two online surveys. The first web-based survey was limited to the PRO-KIND working group members and aimed to finalize the PRO-KIND statements. In parallel, the progress of the PRO-KIND working group was presented and discussed at the annual GKJR Scientific Meetings 2016–2018. Subsequently, all GKJR members were invited to complete a web-based survey. Consensus was considered achieved, when at least 80% of responders supported a statement.

### Consensus treatment plan (CTP) development

The approved statements were integrated into three disease specific CTPs. The PRO-KIND CAPS/TRAPS/ MKD/HIDS expert working group adjusted these in iterative reviews. The final documents were submitted to the GKJR Board for approval.

## Results

### PRO-KIND CAPS/TRAPS/MKD/HIDS working group

The working group commenced its formal work in October 2015 and included 12 experienced German and Austrian paediatric rheumatologists; all experts^1^ in the field of autoinflammatory diseases. All members committed to completing the comprehensive evidence synthesis and iterative statement and CTP development.

### Evidence synthesis, context adaptation and statement development

The updated literature search followed the previously described SHARE search and selection strategy [19] and yielded an additional 25 publications for the timeframe including 19 for CAPS, 4 for TRAPS and 6 for MKD/HIDS; some manuscripts focused on more than one disease. The synthesised literature and current clinical practice of the working group members were reviewed. The 2015 SHARE recommendations for the management of autoinflammatory diseases were adjusted based on new evidence and context. This resulted in 38 drafted statements, of which two were removed due to the lack of consensus. These had focussed on glucocorticoid therapy and stem cell transplantation. A total of 36 statements reached an agreement above 80%; these were nine overarching principles and 27 disease-specific statements including 7 for CAPS, 11 TRAPS and 9 MKD/HIDS (see Additional file 1: Table S1 and Additional file 2: Table S2).

### PRO-KIND CAPS/TRAPS/MKD/HIDS overarching consensus statements

The management of patients with AID requires an interdisciplinary team of generalists and subspecialists including paediatricians, rheumatologists and other specialists including but not limited to ophthalmologists, otolaryngologists, nephrologists and genetic counsellors, as well as physiotherapists, occupational therapists and psychosocial specialists [25]. The impact of AID on individual patients varies widely; close monitoring of disease activity in the individual patient is therefore needed. Treatment aims are early control of diseases activity, prevention of disease and treatment related damage and optimal health-related quality of life (HRQoL). Validated instruments including the patient-reported AIDAI [26] and global assessment scales for physicians and patients/parents [27] should be utilized to determine disease activity and impact in addition to regular assessments and inflammatory markers (see Additional file 1: Table S1). Measures of HRQoL and disease damage should be integrated into care [28, 29].

### PRO-KIND CAPS/TRAPS/MKD/HIDS disease-specific consensus statements

For each disease, specific diagnostic and monitoring statements were developed incorporating the distinct challenges of each condition. The risk of macrophage activation syndrome in MKD/HIDS was based on new evidence [30]. Additional suggestions were made for particularly severe disease. Most importantly, high-level evidence from a randomized controlled trial for the effectiveness of IL-1 inhibition for TRAPS and MKD/HIDS was integrated [27].

### Diagnostic algorithm

**Fig. 2:**
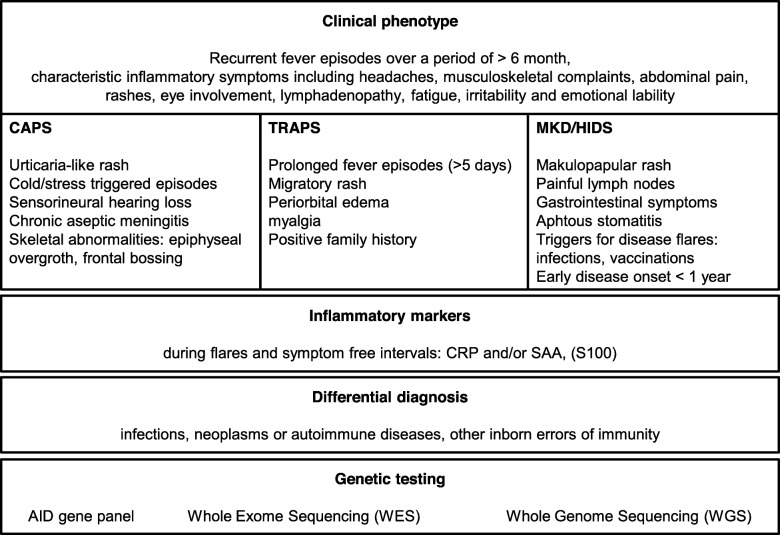
Evidence-based PRO-KIND diagnostic algorithm for CAPS/TRAPS/MKD/HIDS. CRP: C-reactive protein, SAA: Serum-Amyloid A, AID: autoinflammatory disease

The diagnosis of a rare hereditary AID should be considered in patients that experience the following symptoms: recurrent fever, headaches, musculoskeletal complaints, abdominal pain, rash, eye involvement, lymphadenopathy, fatigue and especially increased irritability in children and emotional lability in adults [31] (see Fig. 2). In the presence of these symptoms inflammatory markers including CRP and/or SAA should be determined. The linkage of AID specific clinical symptoms and raised inflammatory markers provides strong evidence of an AID.

In addition typical clinical symptoms for CAPS include urticaria-like rash, cold/stress triggered episodes, sensorineural hearing loss, chronic aseptic meningitis and skeletal abnormalities such as epiphyseal overgrowth and frontal bossing [11]. TRAPS patients may present with prolonged fever episodes of ≥5 days, migratory rash, periorbital edema, myalgia and may have a positive family history [11]. The typical phenotype in MKD/HIDS includes a disease onset at < 1 year of age, gastrointestinal symptoms, painful lymph nodes, aphthous stomatitis, maculopapular rash and disease flares associated with variable triggers such as infections and vaccinations [11]. The differential diagnosis of AIDs has to be thoroughly explored and includes neoplasms, infections, autoimmune conditions and other inborn errors of immunity. Molecular genetic testing should be considered in all patients. Genetic counselling for patients and their families is often required [32]. AID phenotype and genotype can differ substantially, not all genetic variants are pathogenic [31]. The current state of the pathogenicity of a genetic variant can be determined using the infevers database [33]. The presence of a pathogenic variant supports the clinical diagnosis. Potential disease-related damage includes inner ear deafness, vision loss, cognitive impairment, growth retardation, bone and/or joint deformities, osteoporosis, AA Amyloidosis and renal failure.

### CTP development

Treatment strategies for patients with CAPS, TRAPS and MKD/HIDS were derived from the consensus statements. Key principles of these treat-to-target strategies included 1) anchoring individual strategies in disease activity, estimated risk and anticipated trajectories, 2) selecting validated monitoring instruments, 3) defining treatment targets and intervals and 4) providing evidence-based therapies and strategies for adjustment.

After establishing the specific diagnosis, the individual genotype, phenotype and disease activity should be determined. Patients with specific pathogenic mutations should be considered high-risk such as the increased risk of amyloidosis in TRAPS patients with cysteine mutations [30, 31, 34]. Similarly, patients with severe clinical phenotypes, those with frequent inflammatory episodes or those with organ damage at initial presentation are at high risk for adverse outcome. Disease activity and damage should be determined using validated tools. The AIDAI [26] captures disease activity over time including patient-reported frequency and severity of inflammatory clinical symptoms. In addition, inflammatory markers and the physician and patient/parent global assessments (PGA, PPGA) should be determined serially. The ADDI score [29] measures disease-related organ damage.

Complete remission is the key treatment target and defined as absence of clinical symptoms (AIDAI score < 9 [26], PGA and PPGA of 0/10) cm and normal inflammatory markers [27]. If not achievable, minimal disease activity should alternatively be considered. Monitoring intervals of 1–3 months were felt to be appropriate, when patients have active disease; well-controlled disease could be monitored at longer intervals of up to every 6 month.

Overall, treat-to-target strategies guided by multidisciplinary teams have been shown to be highly effective and associated with higher rates of complete remission in patients with CAPS [17]. On demand strategies are considered in some patients with very mild disease and seasonal disease manifestations [35, 36]. IL-1 inhibition has been shown to effectively control inflammation in patients with CAPS, TRAPS and MKD/HIDS [27, 37, 38]. In patients with moderate or severe disease activity, IL-1 inhibition improves or even prevents organ damage [39–41]. Dose adjustments may frequently be required, particularly in children and in patients with severe disease subtypes [17]. Alternatives to IL-1 inhibitions such as TNF-inhibition in TRAPS [42] and IL-6 inhibition in MKD/HIDS [43, 44] need to be further explored. Symptomatic therapies with non-steroidal anti-inflammatory drugs (NSAIDS) or corticosteroids may be effective in some patients.

### CAPS CTP

**Fig. 3:**
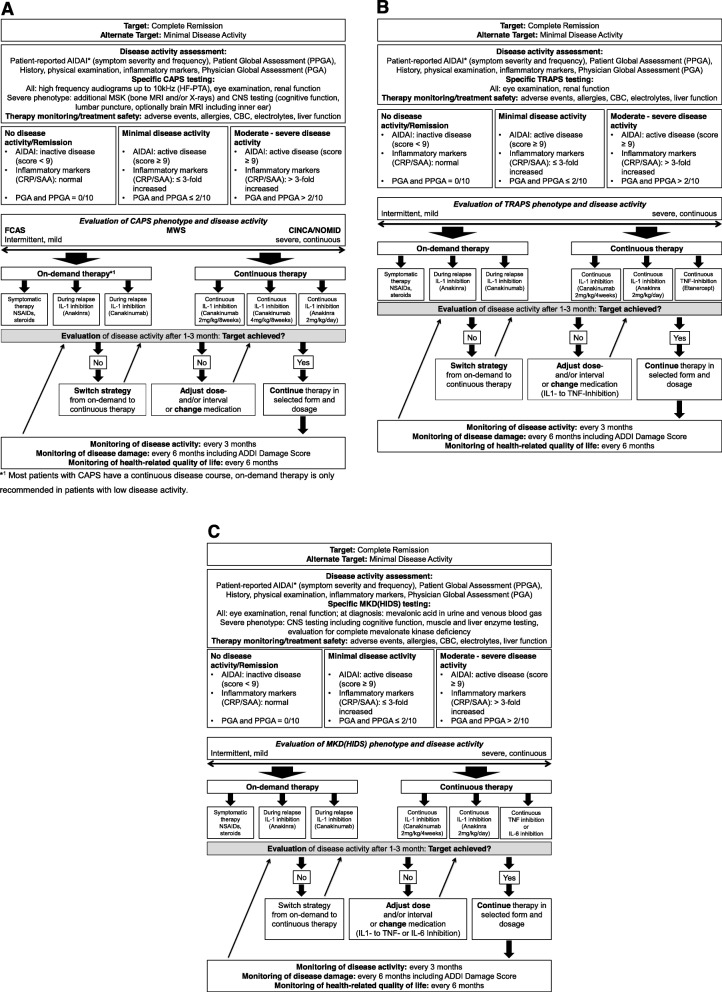
**a** Treat-to-target PRO-KIND Consensus Treatment Plan (CTP) for patients with Cryopyrin-Associated Periodic Syndrome (CAPS)*^1^ Most patients with CAPS have a continuous disease course, on-demand therapy is only recommended in patients with low disease activity. **b** Treat-to-target PRO-KIND Consensus Treatment Plan (CTP) for patients with TNF Receptor-Associated Periodic Syndrome (TRAPS). **c** Treat-to-target PRO-KIND Consensus Treatment Plan (CTP) for patients with Mevalonate Kinase Deficiency Syndrome (MKD). Treat-to-target CTPs for patients with CAPS (**a**), TRAPS (**b**) and MKD/HIDS (**c**) define the treatment targets. After establishing the diagnosis, patients receive either on-demand (only recommended for low disease activity or seasonal disease) or continuous treatment. After 3 months, patients are evaluated for achieving the target. Treatment decisions include continuation, dose adjustments, medication changes, or strategy change. This iterative process aims to ultimately achieve the treatment target of complete remission. Monitoring recommendations and intervals are depicted. AIDAI: AutoInflammatory Diseases Activity Index [26]; ADDI: Autoinflammatory Disease Damage Index for assessment of the extent of organ damage [29]; PGA and PPGA: Physician and patient global assessment using a visual analogue scale. * Definition of remission according to AIDAI [26]

CAPS is a well characterized disease covering a spectrum from mild to severe disease course. Risk factors, disease trajectories and assessment tools for monitoring of disease activity are well studied and validated [10, 45]. The efficacy of IL-1 inhibition was confirmed in randomized clinical trials and high-quality observational studies [41, 46], which led to the approval of canakinumab and anakinra (see Table 1). More recently, 10-year safety data of IL-1 inhibition has become available for CAPS [47]. The effectiveness of treat-to-target studies with individual dose adjustments bares the promise of excellent control of disease activity [17]. Standardized monitoring of disease activity and subsequent dose or strategy adjustments are developed (Fig. 3a). Specific patient groups such as those with a severe phenotype will likely require higher doses to achieve remission [17]. NSAIDs or corticosteroids should be used as add-on therapy in CAPS for symptomatic therapy only [39].

**Table 1:** Current European Medicines Agency and US Food-and-Drug Administration drug approval status for autoinflammatory diseases

Treatment	L	European Medicines Agency (EMA)	Food-and-Drug Administration (FDA)
CAPS			
Canakinumab	1B	CINCA/NOMID, MWS and severe FCAS ≥2 years and ≥ 7.5 kg	FCAS and MWS ≥ 4 years
Rilonacept	1B	none	FCAS and MWS ≥ 12 years
Anakinra	2A	all CAPS patients ≥8 months and ≥ 10 kg	NOMID/CINCA only
TRAPS			
Canakinumab	1B	TRAPS ≥2 years and ≥ 7.5 kg	TRAPS in all adults and children
Anakinra	2B	none	none
Etanercept	2B	none	none
HIDS (MKD)			
Canakinumab	1B	MKD ≥2 years and ≥ 7.5 kg	MKD in all adults and children
Anakinra	2B	none	none
Etanercept	3B	none	none
Adalimumab	3B	none	none
Tocilizumab	4	none	none

The table summarizes indications and specifications for autoinflammatory drug approval as of July 2019. Evidence levels were adapted from the Oxford Centre for Evidence-based Medicine levels of evidence and grades of recommendation [24]: 1B individual randomised controlled trial; 2A systematic review of cohort studies; 2B individual cohort study; 3B, individual case-control study, non-consecutive cohort study; 4 case series

### TRAPS CTP

The disease spectrum in TRAPS is equally broad, however less distinct than CAPS. Genetic variants and other risk factors are important [34]. Several treatment options for TRAPS patients are supported by evidence: IL-1 inhibition with canakinumab is effective and currently the only approved treatment for TRAPS [27] (Table 1). Therapy with other IL-1 inhibitors [36] or the TNF-α decoy receptor etanercept [42] resulted in significant improvement of symptoms and laboratory parameters. In TRAPS, targeting an alternative inflammatory pathway in non- or partial responders may be an important opportunity. Treatment with NSAIDs resulted in symptom relief in 75% of patients with TRAPS, but rarely resulted in effective cessation of inflammation [39]. Corticosteroids may also have an immediate, possibly short-term effect on inflammation in the majority of patients [39]. In TRAPS, treatment decisions should be guided by the initial and subsequent phenotypes, disease activity and risk estimation, standardized monitoring and treatment adjustments are critical to achieve remission (Fig. 3b).

### MKD/HIDS CTP

MKD/HIDS encompasses a wide spectrum of clinical phenotypes from mild to severe and results in diverse symptoms [8]. The effectiveness of interleukin-1 inhibitors in patients with MKD/HIDS was demonstrated and resulted in the approval of canakinumab [27] (Table 1). Additionally, an on-demand approach for anakinra was proposed, which significantly shortened relapses in MKD/HIDS [35]. In individual cases, response to treatment with TNF-α or IL-6 inhibitors has been reported [30, 43]. In severe cases and poor quality of life, allogeneic haematopoietic stem cell transplantation (HSCT) may be considered [48]. The administration of NSAIDs and corticosteroids was shown to improve symptoms [39]. Treatment of patients with MKD/HIDS should follow a treat-to-target approach to find individual treatment strategy, medication and doses (Fig. 3c).

## Discussion

The GKJR PRO-KIND initiative developed the first evidence-based treat-to-target CTPs for autoinflammatory diseases defining treatment targets and integrating patient-centred monitoring of disease activity. The CTPs were established to define standards for the heterogeneous group of autoinflammatory diseases including clinical phenotyping, relevant laboratory tests and genetic testing. They aim to enable and harmonize continuous monitoring of disease activity, provide guidance for clinical examination, laboratory tests and psychosocial care and establish treat-to-target strategies for management to improve health related quality of life and prevent damage.

At diagnosis, the patient’s individual disease activity in addition to the overall AID diagnosis should guide initial management decisions. The disease phenotype is highly variable and can be independent of the presence or type of pathogenic gene variant. In CAPS, the stratification into distinct phenotypes is widely accepted; however, also in patients with TRAPS and MKD/HIDS disease phenotypes can vary dramatically [30, 34]. The individual AID disease activity at diagnosis and at each subsequent evaluation is estimated regarding the extent and severity of clinical symptoms, previous flare duration and frequency plus level of inflammatory markers at time of diagnosis and during flares. These estimates are captured in both patient- and physician derived validated instruments.

Stratified treatment approaches are increasingly made available for childhood rheumatic diseases. Disease subtype, risk factors at onset and disease activity are the foundation for the traditional stratified ACR juvenile idiopathic arthritis (JIA) treatment recommendations [49, 50]. Similarly, the Childhood Arthritis and Rheumatology Research Alliance (CARRA) CTPs for juvenile dermatomyositis stratify approaches based on subtype and disease activity [51, 52]. In contrast, for systemic lupus erythematosus (SLE), type and extent of major organ involvement such as type of lupus nephritis typically drives treatment selections. The SHARE guidelines for childhood-onset SLE recommended different treatment regimens for haematological disease, neuropsychiatric involvement and nephritis further differentiating in histological classes [53, 54].

The PRO-KIND CAPS/TRAPS/MKD/HIDS CTP approach follows the model set by Swart [55] and Consolaro [56] for JIA. The composite patient and physician-partnered disease activity instrument Juvenile Arthritis Disease Activity Score (JADAS) [57] was integrated into clinical care as the anchor of treatment decision making [50]. This has facilitated impactful treat-to-target strategies resulting in better outcomes [55]. The proposed German PRO-KIND consensus statements and resulting CTPs represent an important step forward beyond disease classification-based recommendations for patients with AIDs [19].

The definition of therapeutic targets, the continuous integration of disease activity measurements and the clinical evaluation at defined time points are the basis for the German PRO-KIND CTPs. Therefore, all treatment decisions follow the treat-to-target concept and enable precision health care for children. Continuous disease activity monitoring using evaluated instruments and adjustment of treatment intensity are important to avoid over- or under-treatment. The integration of patient perspective through AIDAI, physician rating of disease activity and assessment of inflammatory markers was utilized in the initial approval studies for IL-1 inhibition in AIDs [26, 27, 46]. While there is no mathematical formula to best integrate the different components, concordant estimates of disease activity clearly support treatment decisions to adjust therapies. Discordant estimates are equally important, as they mandate further evaluations.

Several AID real-life series have focussed on the frequency of achieving remission and the requirement for dose adjustments. Young children with CAPS, in particular those with the severe phenotype NOMID and high disease activity, require much higher starting doses of IL-1-inhibitors and frequent dose increases [17, 58]. This treat-to-target approach successfully controls disease activity and can prevent organ damage in patients with AIDs [59]. In contrast, there is a very mild AID phenotype with limited disease activity: some CAPS patients experience significantly fewer symptoms during warm seasons, TRAPS patients may have very infrequent episodes of clinically active disease [60]. In addition, the risk of organ damage may differ dramatically between AID patients as demonstrated for the risk of hearing loss in CAPS [40].

There are several limitations to the study. 1) The PRO-KIND CAPS/TRAPS/MKD/HIDS expert working group consisted of only 12 members, which limited the scale and scope of contributing experiences. However, the three diseases are bordering on ultra-rare (≤1/1Mio) frequency, limiting the continuous exposure to patients to experts from larger German/Austrian AID centres, all of whom participated in the study. 2) The proposed on-demand strategy for mild phenotypes is supported by low level evidence. However, in clinical practice this approach is utilized in Germany and had to be included as one of the treatment arms in order to allow for inclusion of all patients into CTP-guided care. 3) The generalizability of the German CAPS/TRAPS/MKD/HIDS CTPs is limited to countries with unrestricted, mainly publicly funded access to treatment. However, many of the European countries can potentially follow the proposed paths. The evaluation of the CTPs may enable the community to rapid access to evidence based biologic therapies.

PRO-KIND advances the standardization of diagnosis and management of childhood rheumatic diseases. Publicly available, evidence-based guidance statements and CTPs can decrease variation in care, in particular in rare rheumatic diseases as individual physician preferences may not weigh as heavy as in more common diseases. The engagement and support of the German paediatric rheumatology community was critical to develop the statements, algorithms and CTPs. The integration of the developed framework into clinical practice is the critical next step.

Ultimately the evaluation of these real-life data with comparative effectiveness studies will define if the PRO-KIND approach can optimize health outcomes for children with rheumatic diseases in Germany and beyond.

## Supplementary information

**Additional file 1: Table S1.** PRO-KIND overarching consensus statements for the management of CAPS/TRAPS/HIDS/MKD*^. *^ The PRO-KIND statements were adapted from the SHARE recommendations for the management of autoinflammatory diseases [19]. Evidence levels were adapted from the Oxford Centre for Evidence-based Medicine levels of evidence and grades of recommendation [24]: 1A, Systematic reviews of randomized controlled trials; 1B, individual randomised controlled trial; 2A, systematic review of cohort studies; 2B, individual cohort study; 3B, individual case-control study, non-consecutive cohort study; 4, case series; 5, expert opinions. S, strength of recommendation: A, consistent level 1 studies; B, consistent level 2 or 3 studies or extrapolations from level 1 studies; C, level 4 studies or extrapolations from level 2 or 3 studies; D, level 5 evidence or troublingly inconsistent or inconclusive studies of any level. STIKO: German Permanent Vaccination Commission. ^1^ this recommendation was noticeably modified in comparison with the SHARE recommendations.^2^ regular checks should also be carried out with low or absent disease activity (see treat-to-target). ^3^ in patients with CAPS and HIDS/MKD, severe inflammatory responses have been reported especially to pneumococcal but also meningococcal vaccines [47, 61, 62].

**Additional file 2: Table S2.** PRO-KIND disease-specific consensus statements for diagnosis and management of CAPS/TRAPS/HIDS/MKD ^*. *^ The PRO-KIND statements were adapted from the SHARE recommendations for the management of autoinflammatory diseases [19]. Evidence levels were adapted from the Oxford Centre for Evidence-based Medicine levels of evidence and grades of recommendation [24]: 1A, Systematic reviews of randomized controlled trials; 1B, individual randomised controlled trial; 2A, systematic review of cohort studies; 2B, individual cohort study; 3B, individual case-control study, non-consecutive cohort study; 4, case series; 5, expert opinions. S, strength of recommendation: A, consistent level 1 studies; B, consistent level 2 or 3 studies or extrapolations from level 1 studies; C, level 4 studies or extrapolations from level 2 or 3 studies; D, level 5 evidence or troublingly inconsistent or inconclusive studies of any level. ^1^ this recommendation was noticeably modified in comparison with the SHARE recommendations.

## Data Availability

The datasets used and/or analysed during the current study are available from the corresponding author on reasonable request.

## Abbreviations

ADDI: Autoinflammatory Disease Damage Index
AID: Autoinflammatory Diseases
AIDAI: AutoInflammatory Diseases Activity Index
ALT: Alanin-Aminotransferase
AST: Aspartat-Aminotransferase
CAPS: Cryopyrin-associated periodic syndrome
CARRA: Childhood Arthritis and Rheumatology Research Alliance
CINCA: Chronic Infantile Neurologic Cutaneous Articular Syndrome
CNS: Central Nervous System
CRP: C-reactive protein
CTP: Consensus Treatment Plan
EMA: European Medicines Agency
ENT: Ear, Nose and Throat
FCAS: Familial Cold Autoinflammatory Syndrome
FDA: Food and Drug Administration
FMF: Familial Mediterranean Fever
GKJR: Society for Paediatric and Adolescent Rheumatology (Gesellschaft für Kinder- und Jugendrheumatologie)
HFPTA: High-frequency Pure-tone Audiometry
HIDS: Hyper-IgD-Syndrome
HSCT: Haematopoietic stem cell transplantation
IL-1: Interleukin-1
JADAS: Juvenile Arthritis Disease Activity Score
JIA: Juvenile Idiopathic Arthritis
MKD: Mevalonate Kinase Deficiency Syndrome
MRI: Magnetic Resonance Imaging
MWS: Muckle-Wells-Syndrome
NOMID: Neonatal-Onset Multisystem Inflammatory Disease
NSAID: Non-Steroidal Anti-Inflammatory Drug
PGA: Physician Global Assessment
PPGA: Patient/Parent Global Assessment
PRO-KIND: Projekte zur Klassifikation, Überwachung und Therapie in der Kinderrheumatologie (Projects for the classification, monitoring and therapy in paediatric rheumatology)
SAA: Serum-Amyloid A
SHARE: Single Hub and Access point for paediatric Rheumatology in Europe
SLE: Systemic Lupus Erythematosus
STIKO: German Permanent Vaccination Commission
TNF: Tumour Necrosis Factor
TRAPS: Tumour Necrosis Factor Receptor Associated Periodic Syndrome
VAS: Visual Analogue Scale

## Footnotes

1 Expertise was defined as either strong records of publication in the field of AID and/or head of an AID clinic with more than 25 to 200 patients with rare AIDs per year over the past 5 years.
