# Supplementary material for: Consensus protocols for the diagnosis and management of the hereditary autoinflammatory syndromes CAPS, TRAPS and MKD/HIDS: a German PRO-KIND initiative
Source: Pediatr Rheumatol Online J. 2020 Feb 17;18:17. doi: 10.1186/s12969-020-0409-3 (PMC7027082; doi:10.1186/s12969-020-0409-3)
Supplement: Supplementary file 1 — Additional file 1: Table S1. PRO-KIND overarching consensus statements for the management of CAPS/TRAPS/HIDS/MKD*. * The PRO-KIND statements were adapted from the SHARE recommendations for the management of autoinflammatory diseases [19]. Evidence levels were adapted from the Oxford Centre for Evidence-based Medicine levels of evidence and grades of recommendation [24]: 1A, Systematic reviews of randomized controlled trials; 1B, individual randomised controlled trial; 2A, systematic review of cohort studies; 2B, individual cohort study; 3B, individual case-control study, non-consecutive cohort study; 4, case series; 5, expert opinions. S, strength of recommendation: A, consistent level 1 studies; B, consistent level 2 or 3 studies or extrapolations from level 1 studies; C, level 4 studies or extrapolations from level 2 or 3 studies; D, level 5 evidence or troublingly inconsistent or inconclusive studies of any level. STIKO: German Permanent Vaccination Commission. 1 this recommendation was noticeably modified in comparison with the SHARE recommendations.2 regular checks should also be carried out with low or absent disease activity (see treat-to-target). 3 in patients with CAPS and HIDS/MKD, severe inflammatory responses have been reported especially to pneumococcal but also meningococcal vaccines [47, 61, 62]. [file 12969_2020_409_MOESM1_ESM.docx]

**Table S1**

**PRO-KIND overarching consensus statements for the management of CAPS/TRAPS/HIDS/MKD^*^**

|  | **Level of evidence** | **Strength** | **Agreement**  **PRO-KIND** |
| --- | --- | --- | --- |
| The management of CAPS/TRAPS/HIDS/MKD patients should be guided by a multidisciplinary team in a tertiary care centre with expertise in AID and with access to genetic counselling.* | 5 | D | 91.1 % |
| For patients with CAPS/TRAPS/HIDS/MKD, patient- and family-centred decisions should be made together with a multidisciplinary team.* | 5 | D | 81.4 % |
| The management of patients with CAPS/TRAPS/HIDS/MKD mandates a psychosocial support infrastructure, as the diseases impact health-related quality of life.^#^ | 5 | D | 89.7 % |
| Monitoring of disease activity and damage is important in patients with CAPS/TRAPS/HIDS/MKD and should be done regularly.* | 2B | B | 100 % |
| Frequency of monitoring for clinical, laboratory and imaging diagnostic should depend on disease severity and activity^1^.^#^ | 5 | D | 97.9 % |
| The Autoinflammatory Diseases Activity Index (AIDAI) is a validated tool to assess disease activity and should be utilized in patients with CAPS/TRAPS/HIDS/MKD.^#^ | 2B | B | 93.5 % |
| Aims of the treatment of CAPS/TRAPS/HIDS/MKD include:   - Early and rapid control of disease activity - Prevention of disease and treatment-related damage - Improved participation in daily activities - Improvement of quality of life* | 5 | D | 100 % |
| Physicians should consider other potential causes such as infections, when CAPS/TRAPS/HIDS/MKD patients present with atypical inflammatory episodes.* | 4 | D | 97.8 % |
| Prior to starting biological agents, the required STIKO^2^ vaccinations should be administered, if possible. Currently there are insufficient data available for the safe use of live vaccines during biologic therapy.^#^ | 2B | B | 95.6 % |

Legend: ^*^ The PRO-KIND statements were adapted from the SHARE recommendations for the management of autoinflammatory diseases (19). Evidence levels were adapted from the Oxford Centre for Evidence-based Medicine levels of evidence and grades of recommendation (24): 1A, Systematic reviews of randomized controlled trials; 1B, individual randomised controlled trial; 2A, systematic review of cohort studies; 2B, individual cohort study; 3B, individual case-control study, non-consecutive cohort study; 4, case series; 5, expert opinions. S, strength of recommendation: A, consistent level 1 studies; B, consistent level 2 or 3 studies or extrapolations from level 1 studies; C, level 4 studies or extrapolations from level 2 or 3 studies; D, level 5 evidence or troublingly inconsistent or inconclusive studies of any level.

STIKO: German Permanent Vaccination Commission.

^1^ regular checks should also be carried out with low or absent disease activity (see treat-to-target)

^2^ in patients with CAPS and HIDS/MKD, severe inflammatory responses have been reported especially to pneumococcal but also meningococcal vaccines (47, 61, 62).

* wording modified from SHARE recommendations; ^#^ content modified from SHARE recommendations
