# Supplementary material for: Consensus protocols for the diagnosis and management of the hereditary autoinflammatory syndromes CAPS, TRAPS and MKD/HIDS: a German PRO-KIND initiative
Source: Pediatr Rheumatol Online J. 2020 Feb 17;18:17. doi: 10.1186/s12969-020-0409-3 (PMC7027082; doi:10.1186/s12969-020-0409-3)
Supplement: Supplementary file 2 — Additional file 2: Table S2. PRO-KIND disease-specific consensus statements for diagnosis and management of CAPS/TRAPS/HIDS/MKD *. * The PRO-KIND statements were adapted from the SHARE recommendations for the management of autoinflammatory diseases [19]. Evidence levels were adapted from the Oxford Centre for Evidence-based Medicine levels of evidence and grades of recommendation [24]: 1A, Systematic reviews of randomized controlled trials; 1B, individual randomised controlled trial; 2A, systematic review of cohort studies; 2B, individual cohort study; 3B, individual case-control study, non-consecutive cohort study; 4, case series; 5, expert opinions. S, strength of recommendation: A, consistent level 1 studies; B, consistent level 2 or 3 studies or extrapolations from level 1 studies; C, level 4 studies or extrapolations from level 2 or 3 studies; D, level 5 evidence or troublingly inconsistent or inconclusive studies of any level. 1 this recommendation was noticeably modified in comparison with the SHARE recommendations. [file 12969_2020_409_MOESM2_ESM.docx]

**Table S2**

**PRO-KIND disease-specific consensus statements for diagnosis and management of CAPS/TRAPS/HIDS/MKD ^*^**

|  | **Level of evidence** | **Strength** | **Agreement**  **PRO-KIND** |
| --- | --- | --- | --- |
| **CAPS statements** |  |  |  |
| The following examinations should be performed for all CAPS patients:   - Detailed physical examination including muscle strength, joint status and neurological status, growth and development - Blood tests including AID inflammatory markers C-reactive protein (CRP) and/or serum amyloid A (SAA) - Hearing tests (audiograms) and ophthalmological examination - Urine testing for proteinuria - Disease activity using a validated tool - Health-related quality of life and disease impact on well-being and participation* | 5 | D | 100 % |
| In CAPS patients with severe disease the following examinations should be considered:   - Formal cognitive testing - Lumbar puncture including opening pressure, cell counts and protein - Brain MRI including inner ear imaging - Bone MRI and skeletal X-ray* | 5 | D | 91.1 % |
| For patients with clinically relevant disease activity, IL-1 inhibition is recommended for all CAPS forms and at any age.^#^ | 1B | A | 93.2 % |
| To prevent organ damage, long-term IL-1 inhibition should be initiated as early as possible in patients with clinically relevant disease activity.^#^ | 2B | B | 93.2 % |
| In CAPS, there is no evidence for the effectiveness of DMARDs or other biological therapies than IL-1.* | 2B | B | 88.4 % |
| In addition to adequately dosed IL-1 inhibition, NSAIDs and corticosteroids may be useful symptomatic treatments for short-term use.^#^ | 2B | B | 97.8 % |
| For patients with CAPS, supportive therapies such as physiotherapy, hearing aids and splints are recommended when required.* | 5 | D | 100% |
| **TRAPS statements** |  |  |  |
| The following examinations should be performed for all TRAPS patients:   - Detailed physical examination including growth and development - Blood tests including AID inflammatory markers C-reactive protein (CRP) and/or serum amyloid A (SAA), erythrocyte sedimentation rate (ESR) is optional - Urine testing for proteinuria - Disease activity using a validated tool - Health-related quality of life and disease impact on well-being and participation* | 5 | D | 100 % |
| TNFRSF1A gene variants:  The interpretation of the R92Q and P46L sequence variants can be difficult. These occur at a high frequency in healthy controls; their pathogenic significance remains contentious. However, some individuals develop the clinical phenotype of TRAPS. Fever episodes may be shorter and/or more frequent.* | 2B | B | 97.7 % |
| TNFRSF1A gene variants:  Patients carrying R92Q or P46L variants frequently have milder disease and a better prognosis with improvement over time and a low risk of AA amyloidosis compared to patients with structural TNFRSF1A mutations.* | 2B | B | 90.2 % |
| TRAPS patients with chronic, persistent disease activity have a higher risk of developing AA amyloidosis.* | 2B | B | 100 % |
| NSAIDs may provide symptom relief during inflammatory attacks in TRAPS.* | 2B | B | 100% |
| Short-term glucocorticoids, with or without NSAIDs, may be effective in alleviating inflammatory attacks in TRAPS.^#^ | 2B | B | 97.8% |
| IL-1 inhibition is recommended for TRAPS patients age 2 and older.^#^ | 1B | A | 93.2% |
| TNF-α inhibition with the TNF-α decoy receptor etanercept (off-label use) may be effective in some TRAPS patients; the effect may decrease over time^#^. | 2B | B | 95.5% |
| In patients with frequent TRAPS flares and in those with partially controlled, mildly active disease inflammation between flares, maintenance therapy with IL-1 inhibition or etanercept is recommended to limit corticosteroid exposure.^#^ | 1B-3 | A-C | 95.6% |
| In TRAPS patients, if treatment with the first IL-1 inhibitor at an adequate dose is ineffective or intolerable, a switch to the TNF-α inhibitor etanercept or a different IL-1 inhibitor should be considered. Likewise, if etanercept is ineffective or intolerable, a switch to an IL-1 inhibitor should be considered.* | 2B | B | 91.3% |
| Although a beneficial effect was reported in select cases, TRAPS therapy with a monoclonal anti-TNF-α antibody is not recommended, since it can result in possible paradox hyper-inflammatory reactions. This applies for infliximab, in lower case numbers also for adalimumab.^#^ | 4 | C | 80.0% |
| **MKD/HIDS statements** |  |  |  |
| The following examinations should be performed for all MKD/HIDS patients:   - Detailed physical examination including muscle strength, growth and development - Blood tests including AID inflammatory markers C-reactive protein (CRP) and/or serum amyloid A (SAA), erythrocyte sedimentation rate (ESR) is optional - Ophthalmological examination - Urine testing for proteinuria and hematuria - Disease activity using a validated tool - Health-related quality of life and disease impact on well-being and participation* | 5 | D | 100 % |
| In MKD/HIDS patients with severe disease, the following examinations should be considered:   - Formal cognitive testing - Muscle and liver enzyme testing - Complete neurological examination - Evaluation for complete mevalonate kinase deficiency^#^ | 5 | D | 100 % |
| In MKD/HIDS patients infections and macrophage activation syndrome (MAS) should be considered.^#^ | 2B | B | 93.0 % |
| In MKD/HIDS, NSAIDs may provide symptom relief during inflammatory attacks.* | 2B | B | 100% |
| Short-term glucocorticoids, with or without NSAIDs, may be effective for alleviating inflammatory attacks in MKD/HIDS.* | 2B | B | 95.4% |
| Colchicine and thalidomide are not effective in MKD/HIDS; their use is not recommended.^#^ | 4 | C | 84.6% |
| Short-term IL-1 inhibition may be effective in controlling inflammatory attacks in MKD/HIDS and should be considered to minimize steroid side effects.* | 2B | C | 95.1% |
| With frequent attacks and/or subclinical inflammation between attacks, maintenance therapy with IL-1 inhibition or TNF-α inhibition with etanercept is recommended, and may limit corticosteroid exposure. IL-1 inhibition is recommended for MKD/HIDS patients' age 2 years and older.^#^ | 1B-3 | A-B | 97.7% |
| In MKD/HIDS patients, if treatment with the first IL-1 inhibitor at an adequate dose is ineffective or intolerable, a switch to a different IL-1 inhibitor or another biological agent (including TNF-α or IL-6 inhibition) should be considered. If TNF-α inhibition is ineffective or intolerable, a switch to another biological agent (including an IL-1 or IL-6 inhibiting agent) should be considered.* | 4 | C | 97.4% |

Legend: ^*^ The PRO-KIND statements were adapted from the SHARE recommendations for the management of autoinflammatory diseases (19). Evidence levels were adapted from the Oxford Centre for Evidence-based Medicine levels of evidence and grades of recommendation (24): 1A, Systematic reviews of randomized controlled trials; 1B, individual randomised controlled trial; 2A, systematic review of cohort studies; 2B, individual cohort study; 3B, individual case-control study, non-consecutive cohort study; 4, case series; 5, expert opinions. S, strength of recommendation: A, consistent level 1 studies; B, consistent level 2 or 3 studies or extrapolations from level 1 studies; C, level 4 studies or extrapolations from level 2 or 3 studies; D, level 5 evidence or troublingly inconsistent or inconclusive studies of any level.

* wording modified from SHARE recommendations; ^#^ content modified from SHARE recommendations
